# Plasma Adenosine Deaminase (ADA)-1 and -2 Demonstrate Robust Ontogeny Across the First Four Months of Human Life

**DOI:** 10.3389/fimmu.2021.578700

**Published:** 2021-05-27

**Authors:** Oludare A. Odumade, Alec L. Plotkin, Jensen Pak, Olubukola T. Idoko, Matthew A. Pettengill, Tobias R. Kollmann, Al Ozonoff, Beate Kampmann, Ofer Levy, Kinga K. Smolen

**Affiliations:** ^1^ Precision Vaccines Program, Division of Infectious Diseases, Boston Children’s Hospital, Boston, MA, United States; ^2^ Department of Pediatrics, Harvard Medical School, Boston, MA, United States; ^3^ Division of Medicine Critical Care, Boston Children’s Hospital, Boston, MA, United States; ^4^ Vaccines & Immunity Theme, Medical Research Council Unit The Gambia at the London School of Hygiene and Tropical Medicine, Banjul, Gambia; ^5^ The Vaccine Centre, Faculty of Infectious and Tropical Diseases, London School of Hygiene and Tropical Medicine, London, United Kingdom; ^6^ Department of Pathology, Anatomy, and Cell Biology, Thomas Jefferson University, Philadelphia, PA, United States; ^7^ Telethon Kids Institute, University of Western Australia, Perth, WA, Australia; ^8^ Broad Institute of MIT & Harvard, Cambridge, MA, United States

**Keywords:** adenosine, adenosine deaminase, ontogeny, sex differences, cytokines, chemokines, biomarkers

## Abstract

**Background:**

Human adenosine deaminases (ADAs) modulate the immune response: ADA1 *via* metabolizing adenosine, a purine metabolite that inhibits pro-inflammatory and Th1 cytokine production, and the multi-functional ADA2, by enhancing T-cell proliferation and monocyte differentiation. Newborns are relatively deficient in ADA1 resulting in elevated plasma adenosine concentrations and a Th2/anti-inflammatory bias compared to adults. Despite the growing recognition of the role of ADAs in immune regulation, little is known about the ontogeny of ADA concentrations.

**Methods:**

In a subgroup of the EPIC002-study, clinical data and plasma samples were collected from 540 Gambian infants at four time-points: day of birth; first week of life; one month of age; and four months of age. Concentrations of total extracellular ADA, ADA1, and ADA2 were measured by chromogenic assay and evaluated in relation to clinical data. Plasma cytokines/chemokine were measured across the first week of life and correlated to ADA concentrations.

**Results:**

ADA2 demonstrated a steady rise across the first months of life, while ADA1 concentration significantly decreased 0.79-fold across the first week then increased 1.4-fold by four months of life. Males demonstrated significantly higher concentrations of ADA2 (1.1-fold) than females at four months; newborns with early-term (37 to <39 weeks) and late-term (≥41 weeks) gestational age demonstrated significantly higher ADA1 at birth (1.1-fold), and those born to mothers with advanced maternal age (≥35 years) had lower plasma concentrations of ADA2 at one month (0.93-fold). Plasma ADA1 concentrations were positively correlated with plasma CXCL8 during the first week of life, while ADA2 concentrations correlated positively with TNFα, IFNγ and CXCL10, and negatively with IL-6 and CXCL8.

**Conclusions:**

The ratio of plasma ADA2/ADA1 concentration increased during the first week of life, after which both ADA1 and ADA2 increased across the first four months of life suggesting a gradual development of Th1/Th2 balanced immunity. Furthermore, ADA1 and ADA2 were positively correlated with cytokines/chemokines during the first week of life. Overall, ADA isoforms demonstrate robust ontogeny in newborns and infants but further mechanistic studies are needed to clarify their roles in early life immune development and the correlations with sex, gestational age, and maternal age that were observed.

## Introduction

Early life demonstrates unique immunologic challenges and adaptations related to the transition from an intra-uterine environment and progressive responses to extra-uterine environmental cues (1). This dynamic landscape necessitates age-dependent changes in cellular and soluble factors that shape immunity and that have yet to be fully characterized (1–7). Given that infancy is the time of receipt of most vaccines coupled with the heavy burden of early life infection, and a period of profound changes in the immune system, a better understanding of immune ontogeny in human newborns is essential.

Among the soluble immunoregulatory proteins of human plasma are adenosine deaminases (ADAs) -1 and -2. ADA1 (41kDa) is encoded by the *ADA* gene on chromosome 20q13.12 (OMIM 608958 or Entrez Gene ID 100) and is produced by all cells (8, 9). While the intracellular role of ADA1 has been established, this enzyme also has extracellular roles (10–13), including formation by ADA1 (or ecto-ADA) of a ternary complex with CD26 and A2a receptors bridging two different cells as a co-stimulatory molecule that impacts T-cell proliferation (14, 15). ADA1 converts adenosine, an endogenous purine metabolite that acts *via* leukocyte purine receptors to suppress pro-inflammatory and Th1-polarizing responses, to inosine, which is immunologically inert (16–19). ADA1 also has roles in enhancing T-helper 2 (Th2) immunity *via* adenosine receptors (20). ADA1 deficiency impairs thymocyte development and B-lymphocyte immunoglobulin production (21) resulting in severe combined immunodeficiency (22).

ADA2 has a higher *Km* for adenosine (23, 24) and is thereby less enzymatically active than ADA1. While residual ADA2 activity ADA2 can be measured in patients with ADA1 deficiency (23, 25), its important roles in immunity has previously been under-appreciated. ADA2 (57kDa) is encoded by the *CECR1* (*ADA2)* gene on chromosome 22q11.1 (OMIM 607575 or Entrez Gene ID 51816) and is produced by activated monocytes, macrophages, and dendritic cells (DCs) (8, 9). Independent of its enzymatic activity, ADA2 modulates immunity *via* binding cognate receptors on immune cells (14, 26, 27). ADA2 also induces monocyte differentiation to macrophages in T-cell co-cultures (14). ADA2-deficient cells are unable to differentiate into M2/pro-resolution macrophages (24, 28, 29) suggesting that ADA2 directs differentiation of macrophages towards an anti-inflammatory phenotype. First described in 2014 (30, 31), ADA2 deficiency (DADA2) presents with heterogeneous manifestations of which vascular inflammation is predominant (32–52). Patients with ADA2 deficiency and vasculitis often have missense mutations with at least 3% residual activity, whereas complete loss of function was associated with pure red cells aplasia and bone marrow failure (39). ADA2 binds to neutrophils, monocytes, NK cells, and B cells (27), and patients with ADA2 deficiency can present with inflammatory conditions and altered distribution of immune cell subsets and immunoglobulin levels (32, 35, 39–41, 47, 50, 51). Overall, ADA2 is a protein of relevance to the human immune system whose expression in early life has been incompletely characterized.

We have previously demonstrated that adenosine inhibits TLR-induced production of TNFα but not IL-6 and that pre-incubation of cord blood mononuclear cells with recombinant ADA1 (rADA1) enhances TLR-mediated TNFα production (16). Moreover, in a small cohort (n = 4-12 per group), newborns exhibit lower plasma concentrations of extracellular ADA1 compared to adults, resulting in elevated newborn plasma adenosine concentrations and a Th2/anti-inflammatory bias (3, 16). However, these studies did not evaluate plasma ADA concentrations within the first month of life, nor did they assess whether plasma concentrations of these enzymes correlate with plasma cytokine and chemokine concentrations. Thus, how ADA1, ADA2, and total ADA change during the first week of life and subsequent months of life, when the immune system of neonates and infants undergo dramatic immunologic changes, are most susceptible to infection, and receive the greatest number of vaccines, is still unknown.

Partnering as international collaborators *via* the Expanded Program on Immunization Consortium (EPIC), we conducted the EPIC002 study, a prospective study to characterize immunologic biomarkers in newborn infants followed across a four-month period (53). We measured ADA1 and ADA2 in infant plasma to determine age-dependent changes in early life. We presented higher resolution data on ontogeny of not only total ADA, but also ADA1 and ADA2 during the first week of human life in a large cohort of infants (N = 540 participants). We investigated the ontogenic patterns in plasma ADA1 and ADA2 concentrations across five Gambian ethnic sub-groups. We explored whether demographic factors such as sex, gestational age, and maternal age were associated with distinct ADA concentrations in infant plasma. Finally, we assessed whether plasma ADA1, ADA2, and total ADA correlated with plasma concentrations of cytokines and chemokines during the first week of life. Overall, our study revealed that these immune-regulatory proteins demonstrate robust changes across the first week and months of life and correlate with plasma cytokine and chemokine concentrations, suggesting a functional role for ADAs in human immune ontogeny.

## Methods

### Study Design and Sample Collection

The Expanded Program on Immunization Consortium (EPIC) study 002 (EPIC002) clinical protocol has been previously described (53), and the study is registered on clinicaltrials.gov as NCT03246230. The study’s primary goal was to assess vaccine immunogenicity in newborns in 4 different vaccine groups (no vaccines at birth (i.e., delayed immunization), Hepatitis B vaccine (HBV) alone at birth, BCG alone at birth, and both (HBV and BCG) at birth; with n = 180 per group).

In brief, mothers and their newborns were consented and enrolled at time of delivery at the Medical Research Council (MRC) Unit at the London School of Hygiene and Tropical Medicine in The Gambia. Mothers were enrolled only if they were above the age of 18 years old, HIV-negative, had no history of tuberculosis (TB) diagnosis in the mother or family member in the past six weeks prior to enrollment using an electronic case report form (eCRF) and were Hepatitis B-negative; additional maternal exclusion criteria included severe pre-eclampsia and/or physician assessment of high-risk pregnancy such as recurrent early neonatal death. Newborns were included only if gestational age >36weeks (as determined by Ballard scoring), if Apgar scores at 5th minute >8, if birth weight >2.5 kilograms (kg). Infants with macrosomia (birth weight >4kg) with existing risk factors such as major known congenital malformation or abnormal exam as determined by physician assessment at birth were also excluded.

Peripheral blood samples were collected using sterile sodium heparin tubes (Becton Dickinson) from infants at four time points. The first sample, Visit 1, was collected within the first 24 hours of life (Day of Life (DOL)0); Visit 2 sample was collected at either DOL1, DOL3, or DOL7; Visit 3 sample was collected at DOL30 and finally Visit 4 sample was collected at DOL128. Visit 3 and Visit 4 were collected in only 540 of the 720 maternal-newborn pairs enrolled, specifically, only in the HepB, BCG, HepB plus BCG at birth groups, per protocol (53) due to limitations in cost. Hence only the 540 infants for whom plasma biosamples were available for ADA assay at all timepoints (i.e., Visits 1 thru 4) were included in the ADA ontogeny sub-analysis that is the focus of this report. Plasma samples were processed for analysis as we have previously described (4). All plasma samples were stored at -80°C until use. Local and International (collaborator) Ethics and/or IRB committees approved the clinical protocols.

### Adenosine Deaminase Assay Methods

#### Reagents

Adenosine Deaminase Assay Kit, including: ADA Assay reagent kits [cat. # DZ117A], ADA calibrator [cat. # DZ117A-Cal], and Quality Controls [cat. # DZ117A-Con] (Diazyme Laboratories, Poway, CA, USA) and Erythro-9-(2-hydroxy-3-nonyl) adenine (EHNA) [cat. # 1261] (Tocris Bioscience, Bristol, UK).

#### ADA Chromogenic Assay

ADA1 and ADA2 concentrations in plasma samples were measured with an ADA Assay Kit per the manufacturer’s instructions (Diazyme Laboratories, Poway, CA, USA), run in duplicate with or without EHNA (20 µM) on a 384 well plate. ADA2 is not EHNA sensitive, and thus activity in EHNA-containing wells was considered to reflect ADA2 activity. ADA1 concentration was calculated by subtracting ADA2 concentration from total ADA concentration.

#### ADA Chromogenic Assay Analysis

The plate was read on an Infinite M Plex (Tecan, Mannedorf, Switzerland), programmed to run a kinetic cycle at 37°C with absorbance readings at 550nm performed every 5 minutes over 1 hour.

### Cytokine/Chemokine Methods

#### Reagents

Dulbecco’s phosphate-buffered saline (dPBS, cat 14190), Corning CellBIND^®^ 384 well plates (cat # CLS3764), and Milliplex Human Cytokine/Chemokine MAGNETIC BEAD Premixed 41 Plex Kit. (Millipore HCYTMAG-60K-PX41).

#### Cytokine/Chemokine Assay

Plasma was diluted 1:2 in dPBS prior to use. Diluted plasma samples and the Milliplex 41-plex kit manufacturer provided standards and quality controls were then assayed and results were obtained with a Flexmap 3D system with Luminex *xPONENT* software (both from Luminex Corp.; Austin, TX, USA). Cytokine concentrations were determined using Milliplex Analyst software (version 5.1.0.0).

### Statistical Methods

For the analysis of ADAs, the change in absorbance between individual time points was calculated and averaged over all time points to obtain the rate of absorbance change for each sample. The rates were averaged between duplicates. Each plate was converted to concentration in units per liter (U/L) using a log-standard curve, and plates with randomized samples were normalized to the overall mean and standard distribution using a universal standard sample. ADA1 concentration was imputed by subtracting the concentration of ADA2 from the total ADA concentration measured for each sample.

Observations were log_10_-transformed to generate a data set of approximate normality, and fold-change calculated in relation to DOL0 (Visit 1). Longitudinal statistical comparisons employed ANOVA for non-repeated measurements with post-hoc analysis using Welch’s t-test. Gaussian-distributed data was modeled with generalized estimating equations (GEE), using an identity link function and exchangeable covariance structure for longitudinal comparisons with repeated measures. GEE significance was calculated from the Wald statistic after performing deviance analysis against a null model (54). Comparisons between demographic and physiological variables like biological sex, heart rate, and gestational or maternal age were analyzed using untransformed data (U/L) and rank sum Wilcoxon and Kruskal-Wallis tests (55) to allow for comparison with previously published data (3, 56–60) and hospital-based tests on absolute plasma ADA1, ADA2, and total ADA concentrations. For tables and graphs presenting absolute activity concentrations, the median and the interquartile range (IQR) were used for descriptive statistics (61).

For cytokine/chemokine analysis, the *xPONENT* software files were processed using the *drLumi* R package. The standard curves were fitted using a 4-parameter logistic, 5-parameter logistic, and exponential function by the *drLumi:*:scluminex() function. The best-fit curve was used for each cytokine. The lower and upper limits of detection were set as the lowest and highest concentration of the standard curve, respectively. Analytes whose concentration could not be estimated were imputed to either the lower (LLOQ) or upper (ULOQ) limit of quantification for that plate/analyte. Samples that had all analytes below the lower limit of detection were excluded from the final analysis. The raw cytokine or chemokine values were then log_10_-transformed to achieve a Gaussian distribution. ComBat (62) (sva R package) was then used to further normalize across plates based on plate-specific biases as determined by PCA plots. Correlation coefficients between analytes and ADA concentrations during the first week of life were calculated using Spearman’s rho, p-values were determined by R function cor.test, and adjusted using the Holm-Bonferroni method.

Clinical metadata was evaluated based on potential interactions with adenosine and ADAs and biomarkers in general. For example, inhibition of TNFα by adenosine is thought to be cardio-protective both for ischemic heart disease and congestive heart failure (63–66). Gestational age (GA) correlates with biomarkers such as hemoglobin and iron (67–70) and advanced maternal age, defined as age ≥35 years old, can be associated with high-risk pregnancy and inflammatory states like pre-eclampsia (71–73), where ADAs may be altered (74). Thus, variables such as heart rate, gestational age, and maternal age were first analyzed as continuous variables (data not shown) prior to categorization. Definition of categories:

1) *Maternal age* categories were based on standard age group of mother in the Morbidity and Mortality Weekly Report by the United States Center for Disease Control or other Demographic and Health Surveys (75).
2) *Gestational age* (GA) is categorized based on the American College of Obstetricians and Gynecologists (76).
a. Early term: 37 0/7 weeks through 38 6/7 weeks,
b. Full term: 39 0/7 weeks through 40 6/7 weeks,
c. Late term: 41 0/7 weeks through 41 6/7 weeks.

Statistical analyses employed R version 3.6.3, using package versions ggpubr_0.3.0 and gee_4.13-20, for ANOVA/Wilcoxon/Kruskal-Wallis tests and GEE, respectively. Significant p-values depicted as *= p<0.05, ** = p<0.01, *** = p<0.001; **** = p<0.0001; ns = not significant.

## Results

### Baseline Characterization of Study Participants

**Figure 1:**
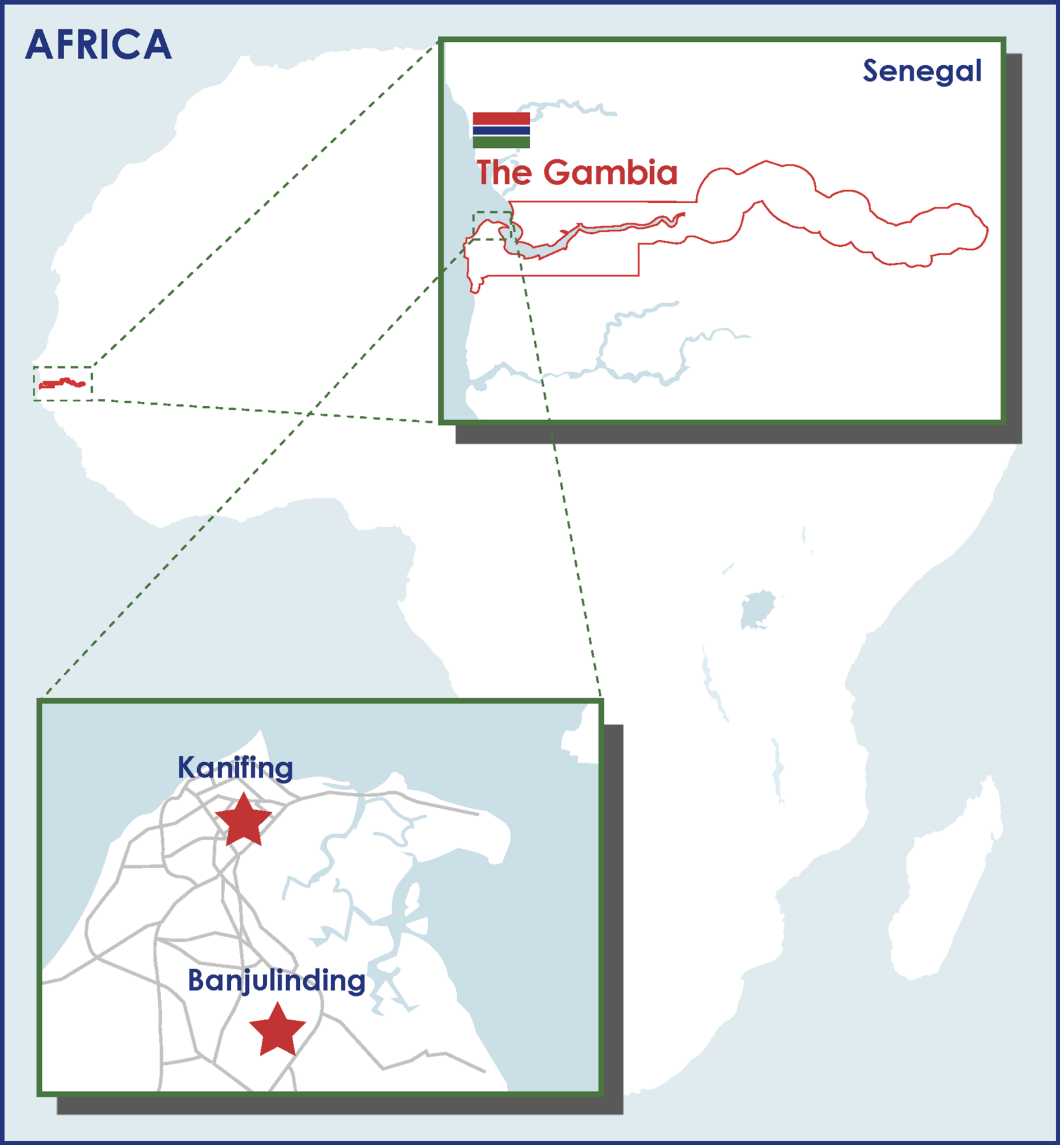
Geographical distribution of the two recruitment sites for the EPIC002 study in The Gambia.

540 Gambia mother-newborn pairs enrolled in the EPIC002 cohort were followed for 128 days and were included in our analysis. As shown in **Table 1**, the majority of mothers (30.7%) were 25-29 years old, followed by age 20-24 years old (22.2%) and then age 30-34 (19.1%). A few preterm newborns (n=4, 0.7%), defined as <37 weeks gestation, were enrolled but the majority (87.2%) of newborns were early term (≥37 weeks to <39 weeks gestation) or full term (≥39 weeks to <41 weeks gestation). Participants were recruited from 2 sites (**Figure 1**), and the Mandinka, Jola, and Fula groups made up the majority of ethnic sub-groups (78.2%). There was an approximately equal ratio of male and female newborns enrolled (49.1% female, 50.9% male) and the average birth-weight was 3.2 kg. Initiation of breastfeeding was 87.5% at delivery and continued after the first day of life (>98%) until four months of age for infants in this cohort.

**Table 1:** Characteristics of EPIC002 study participants.

Characteristics	Frequency (N)	Percent (%)
**Sex of the newborn:**		
Female/Male	265/275	49.1/50.9
**Birth weight (kg)**		
Avg ( ± SEM)	3.163 ± 0.017	
**Maternal age (years)** ^**a**^:		
15 - 19	23	4.3
20 - 24	120	22.2
25 - 29	166	30.7
30 - 34	103	19.1
35 - 39	89	16.5
40 – 45	39	7.2
**Gestational age** (based on Ballard scoring):		
Preterm (<37 weeks)	4	0.7
Early term (≥37 – <39 weeks)	132	24.4
Full term (≥39 – <41 weeks)	339	62.8
Late term (≥41 weeks)	65	12
**Newborn ethnic sub-group:**		
Mandinka	269	49.8
Jola	83	15.4
Fula	70	13
Wolof	50	9.3
Serahule	22	4.1
Others	46	8.5
**Frequency of breastfeeding**		
Visit 1 (Yes/No)	454/65	87.5/12.5
Visit 2 (Yes/No)	519/6	98.9/1.1
Visit 3 (Yes/No)	520/6	98.9/1.1
Visit 4 (Yes/No)	510/5	99.0/1.0

^a^In EPIC002, all maternal participants are age 18 and above.

### Ontogenic Changes in ADA1, ADA2, and Total ADA Across the First Four Months of Life

**Figure 2:**
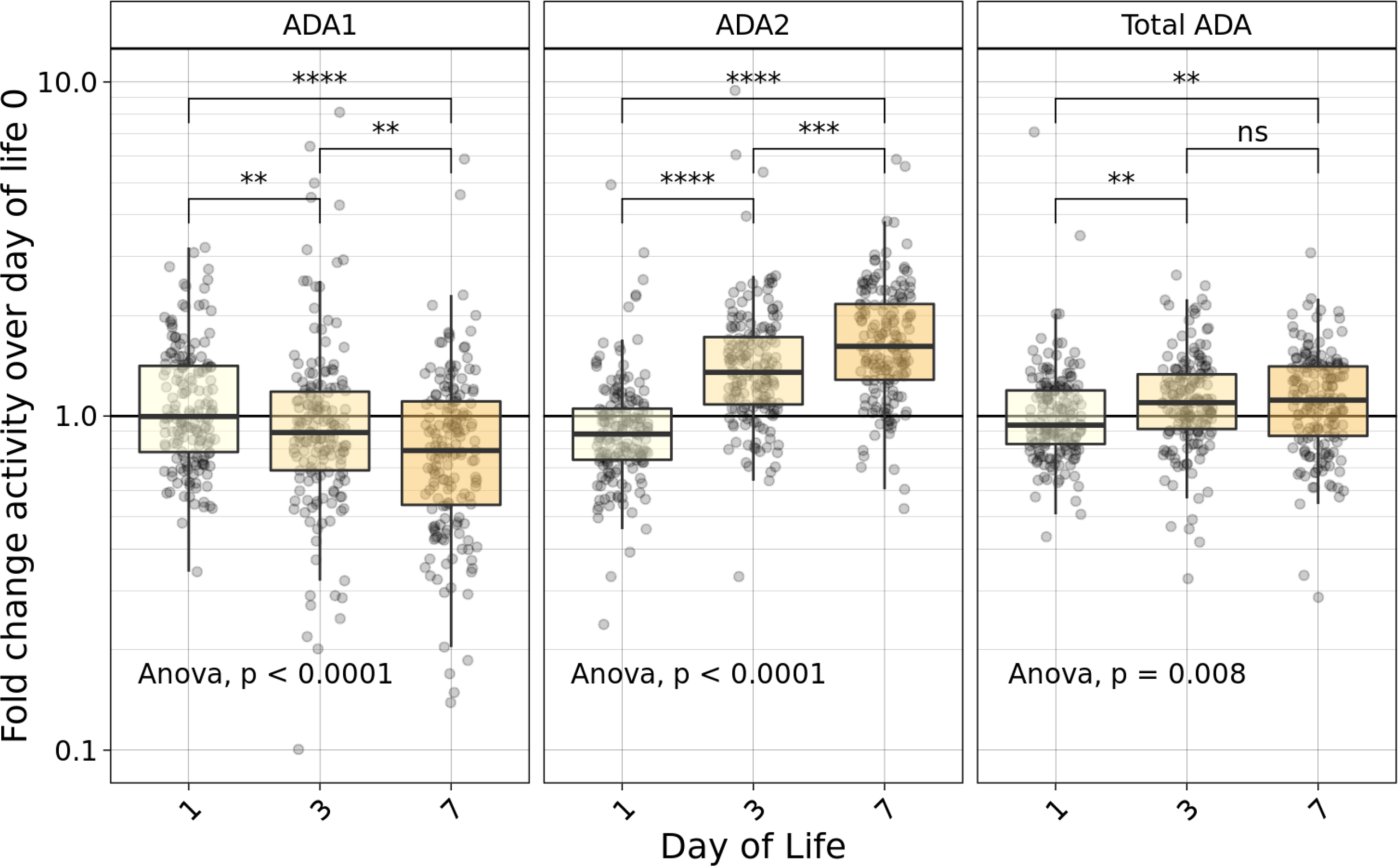
Fold-change relative to DOL0 of ADA concentrations measured in plasma during the first week of life varied based on type of ADA measured. Fold-change of ADA1 (left), ADA2 (middle) and Total ADA (right) plasma concentrations relative to the day of life (DOL) 0 in the Gambian cohort demonstrated an increase in relative plasma ADA2 and total ADA, as well as a decrease in ADA1 across the first week of life (n =168-173/group). Statistical analyses employed ANOVA followed by Welch’s t-test for pairwise comparisons. Significant p-values depicted as **p<0.01, ***p<0.001; ****p<0.0001; ns, not significant.

We measured the concentrations of plasma ADA1, ADA2, and total ADA during the first week in the Gambian cohort. There was a significant fold change (p ≤ 0.0001) decrease in ADA1, and an increase in ADA2 and total ADA (**Figure 2**). Specifically, while plasma concentrations of ADA1 decreased by 23% (from 4U/L at DOL0 to 3.1U/L at DOL7), concentrations of both ADA2 (from 2.6U/L at DOL0 to 4U/L at DOL7), and total ADA (from 6.7 U/L at DOL0 to 7.5U/L at DOL7), increased by 54% and 12%, respectively, across the first week of life (n =168-173 per group) (p<0.01). However, there was no significant difference in total ADA from DOL3 to DOL7.

**Figure 3:**
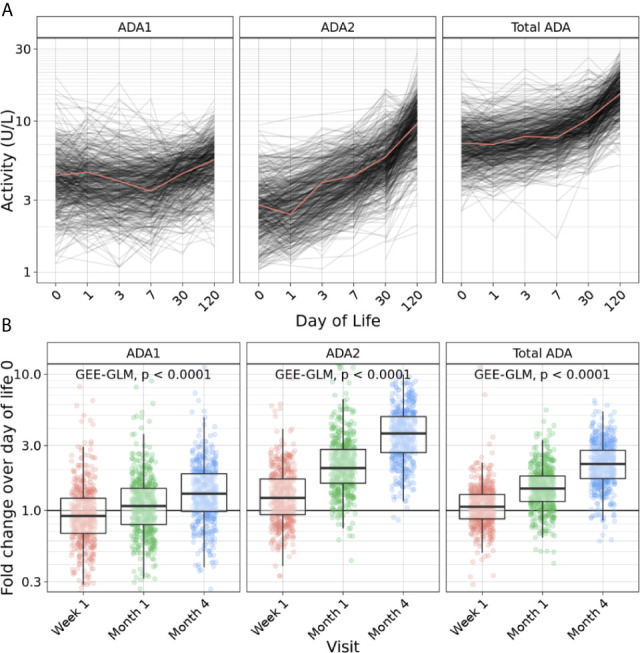
Plasma ADA concentrations increased across the first four months of life. **(A)** Concentration of ADA1 (left), ADA2 (middle) and Total ADA (right) during the first 128 days of life in the Gambian cohort showed an increase of ADA1, ADA2 and total ADA concentrations across the first four months of life (n = 540 participants). **(B)** Log_10_ fold-change of ADA1 (left), ADA2 (middle) and Total ADA (right) relative to day of life (DOL) 0 in the Gambian cohort demonstrated an increase of ADA1, ADA2 and total ADA concentrations during the first four months of life (n = 491-511) (p<0.0001). Statistical analyses fit a GEE-GLM to log_10_ (activity) with Visit as a predictor variable, using Gaussian distribution, identity link function, and exchangeable covariance structure. Deviance analysis was conducted by comparing GEE to a null model, and p-values were found using the Wald statistic.

Next, we investigated ADA isoforms beyond the first week of life by measuring the relative ADA concentration (**Figure 3A**) and the fold change compared to DOL0 (**Figure 3B**) of ADA1, ADA2, and total ADA during the first four months. Interestingly, the concentration of all ADA subtypes (total ADA, ADA1, and ADA2) increased over the first four months of life consistent with an overall increase in plasma ADA concentrations with age (p<0.0001) (n = 491-511) (**Figure 3**). Since total ADA is defined as the sum of ADA1 and ADA2, we explored the ratio of ADA2 relative to ADA1 concentration across time. From DOL3, the ratio of ADA2/ADA1 increased (p<0.0001), suggesting that elevated ADA2 activity may contribute to the total ADA measured (**Supplementary Figure 1**).

### Association of Extracellular Plasma ADAs Concentration and Demographic Factors

First, we explored associations between ADA concentrations and physical exam findings at birth to look for confounders. No significant differences were observed in concentrations of ADA1, ADA2, or total ADA based on respiratory rate, heart rate, weight, length, head circumference, or temperature at birth (data not shown). No difference in ADA concentrations after the first week of life were noted in The Gambian ethnic sub-groups (**Supplemental Figure 2**).

**Figure 4:**
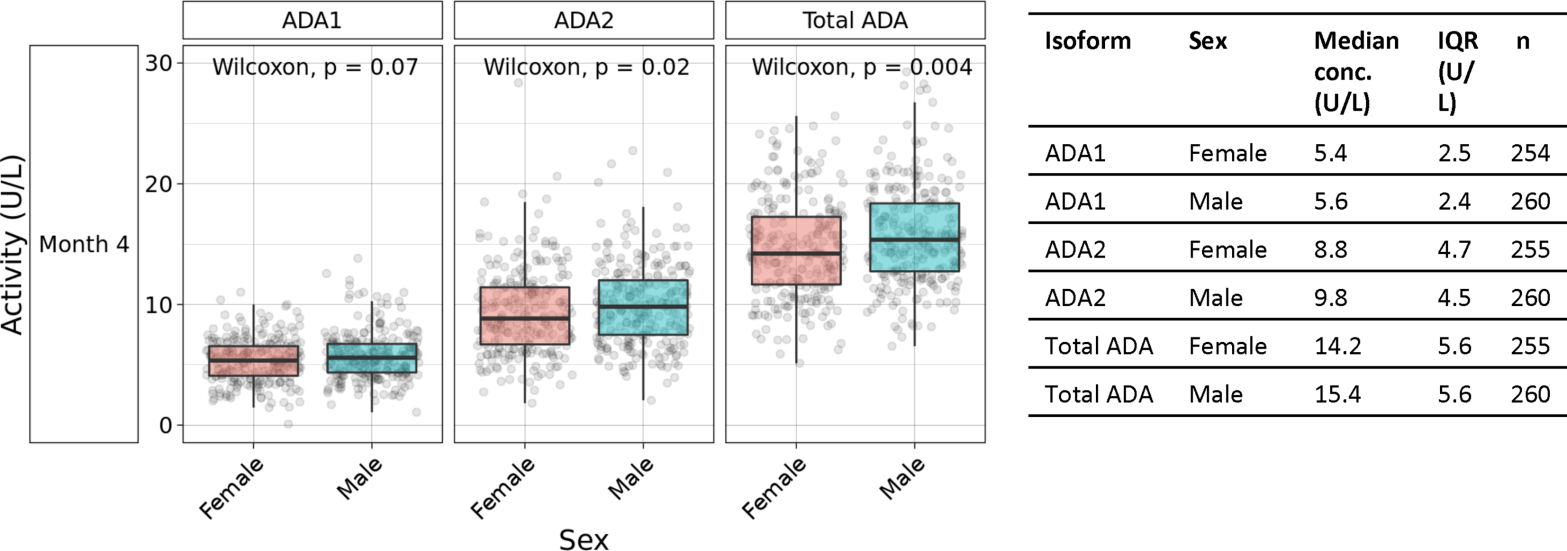
Males exhibited higher plasma ADA2 concentrations at four months of age. Measurement of plasma ADA1, ADA2, and Total ADA in biosamples in the Gambian cohort demonstrated greater concentrations of ADA1 (p=0.07), ADA2 (p=0.02) and Total ADA (p=0.004) in males than females during the first four months of life (n =254-260 per group). Statistical analyses employed Wilcoxon rank sum test.

Sex-based differences were observed in plasma concentrations of ADA2 and total ADA, but not ADA1. At 4 months of life, males demonstrated 11% higher median plasma ADA2 concentrations (9.8 U/L *vs.* 8.8 U/L) and 8.5% higher total ADA (15.4 U/L *vs.* 14.2 U/L), whereas ADA1 was 3.7% higher (5.6 U/L *vs.* 5.4 U/L) (**Figure 4**). This pattern was significant for ADA2 (p=0.02) and total ADA (p=0.004) (n =254-260 per group). Furthermore, sex-specific differences in ADA concentrations were not observed in the first 30 days of life but only noted at four months of age (**Supplementary Figure 3**).

**Figure 5:**
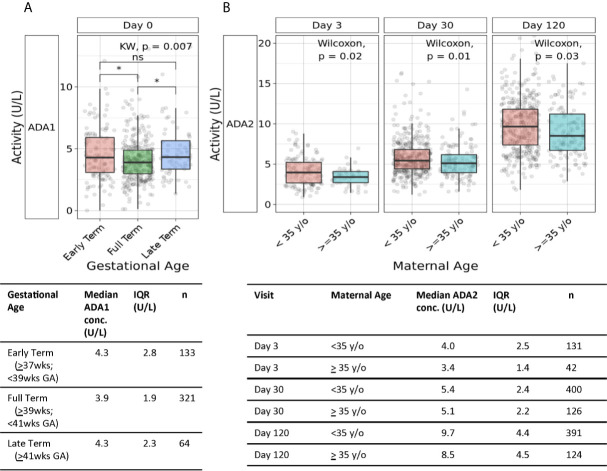
Association of gestational age and maternal age with plasma ADA concentrations. **(A)** Both early and late gestational age were associated with higher neonatal plasma concentrations of ADA1. Plasma concentrations of ADA1 at Day 0 in the Gambian cohort were significantly elevated in both early and late-term versus to full-term newborns (n = 133 early-term, n = 321 full-term, n = 64 late-term)(*p<0.05; ns, not significant). Statistical analyses employed Kruskal-Wallis and Wilcoxon for post-hoc. **(B)** Greater maternal age was associated with lower ADA2 concentrations during the first four months of life. Concentrations of plasma ADA2 in the Gambian cohort were significantly elevated at Day of Life 3 (p=0.02), 1 month (p=0.01), and 4 months (p=0.03) (n = 131- 400 infants of mothers <35 years of age, n = 42-126 infants of mothers ≥35 years of age). Statistical analyses employed Wilcoxon rank sum test.

Plasma ADA1 concentrations were elevated in early term (4.3 U/L) (p<0.05) and late term newborns (4.3 U/L) (p<0.05) compared to full term (3.9 U/L), exhibiting a 10% increase (**Figure 5A**) (n=64-321 per group). At 1 week of life and then 4 months of life, when ADA2 becomes more prominent (**Supplemental Figure 1**), early gestation age was also associated with increased ADA2 and total ADA (but not ADA1) (**Supplemental Figure 4**). Our study was not designed to assess any possible effects of prematurity (GA <37 weeks) on ADA concentrations for which sample size was low (n=4).

Advanced maternal age, defined as age ≥35 years old, can be associated with high-risk pregnancy and inflammatory states like pre-eclampsia. Accordingly, we assessed whether advanced maternal age (n=124-126) was associated with differences in neonatal plasma ADA1 and ADA2 concentrations. We observed lower ADA2 concentrations in infants born to mothers of advanced maternal age, at DOL3 (3.4U/L *vs.* 4U/L) (p=0.02), 1 month (5.1U/L *vs.* 5.4U/L) (p=0.01), and 4 months of life (8.5U/L *vs.* 9.7U/L) (p=0.03) (**Figure 5B**). A similar observation of lower total ADA concentration in newborns of women ≥35 years of age was significant at DOL3 (p=0.006) and demonstrated strong trends at 1- and 4- months (p = 0.06 and 0.07, respectively) (**Supplementary Figure 5**).

### Correlation With Plasma Cytokines or Chemokines and ADA Subtypes

**Figure 6:**
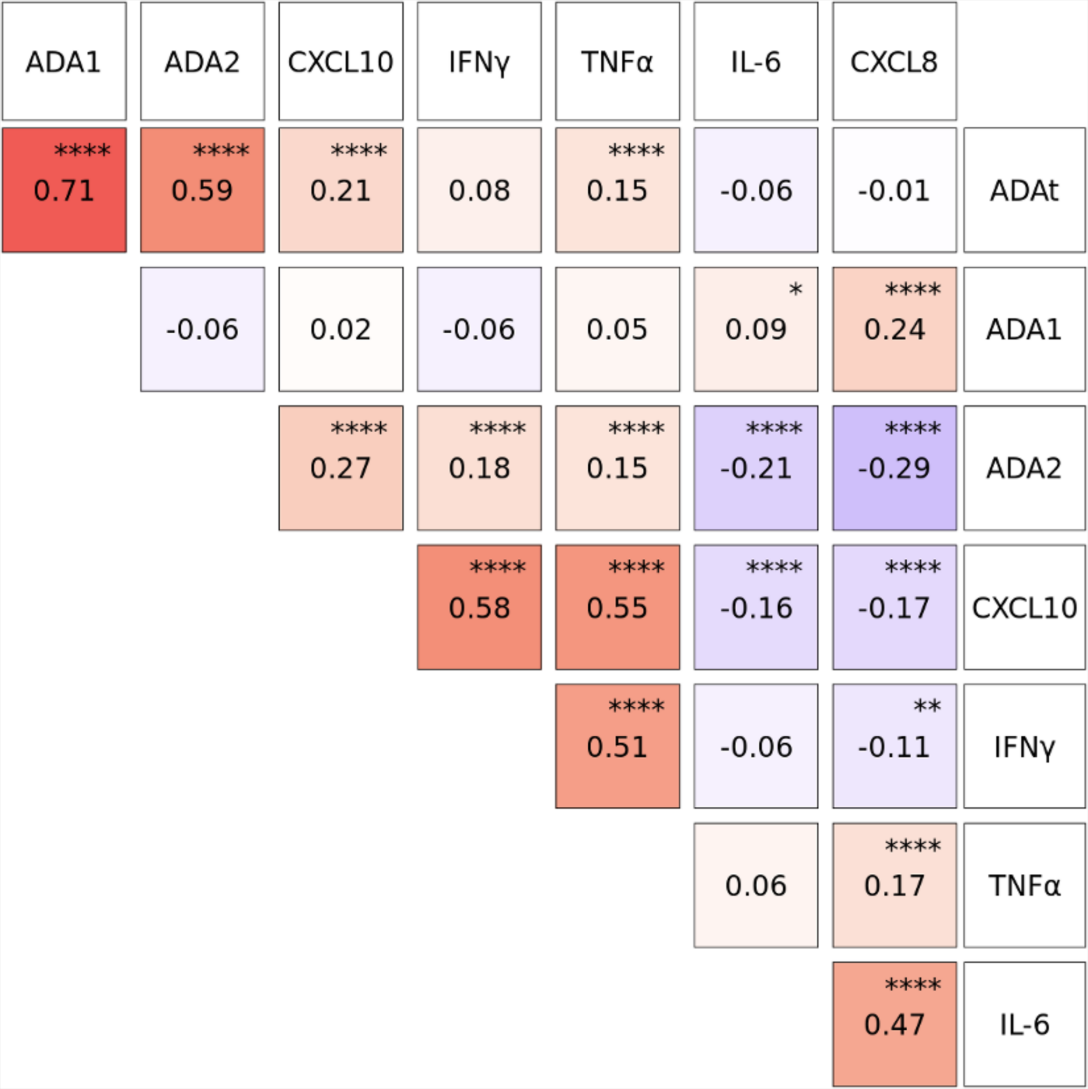
Plasma concentrations of CXCL10, IFNγ and TNFα were positively correlated with Total ADA (ADAt) and ADA2 during the first week of life. Log_10_-transformed plasma cytokine and chemokine concentration were pooled from Visit 1 and Visit 2 during the first week of life (n= 1027-1044 based on Visit 1 and Visit 2). Correlation coefficients between analytes were calculated using Spearman’s rho and plotted using the GGally package in R. Total ADA (ADAt) and ADA2, but not ADA1, were positively correlated (red) with CXCL10, IFNγ and TNFα. ADA1 was positively correlated (red) with IL-6 and CXCL8 while ADA2 was negatively correlated (blue) with IL-6 and CXCL8. P-values were determined by R function cor.test, and adjusted using the Holm-Bonferroni method. Significant p-values depicted as *p<0.05, **p<0.01, ****p<0.0001.

We measured plasma concentrations of several cytokines, including IFNγ, TNFα, and IL-6, as well as chemokines such as CXCL8 and CXCL10 at DOL0, 1, 3, and 7. Robust rho values were observed for the correlation between Th1-polarizing cytokines: CXCL10 (aka IP-10) with IFNγ (r=0.58) and TNFα (r=0.55), as well as IFNγ with TNFα (r=0.51) (p<0.0001). As expected, total ADA correlated with the concentration of ADA1 (r=0.71) and ADA2 (r=0.59) (p<0.0001). In assessing correlations between plasma ADAs and cytokines or chemokines, we noted that total ADA was significantly correlated with CXCL10 (r=0.21) and TNFα (r=0.15) (p<0.0001) during the first week of life (**Figure 6**) but not with IFNγ, IL-6, or CXCL8. ADA1 correlated positively with CXCL8 (r=0.24) (p<0.0001) and IL-6 (r=0.09)(p<0.05), while ADA2 correlated positively with CXCL10 (r=0.27), IFN-γ (r=0.18), and TNFα (r=0.15) (p<0.0001, but negatively with IL-6 (r=-0.21) and CXCL8 (r=-0.29) (p<0.0001).

## Discussion

To our knowledge, our study is the first that has explored the ontogeny of plasma ADA1, ADA2, and total ADA across the first week of human life. We characterized changes in plasma ADA1, ADA2, and total ADA over the first four months of life and defined novel correlations with sex, gestational age, and maternal age. We observed significant positive correlations between plasma concentrations of ADA2 and those of CXCL10, IFNγ, and TNFα; in addition to negative correlations with IL-6 and CXCL8.

In depth characterization of the distinct immune ontogeny of human newborns is important to inform development of better agents and approaches to prevent, diagnose, or treat infection in early life (6, 20). We recently demonstrated that dynamic age-dependent molecular and cellular changes occur in the first week of life using a systems biology approach (4). Building on our previous work that suggested total ADA increases over the first year of life (3), we presented higher resolution data on ontogeny of total ADA, ADA1, and ADA2 during the *first week of life* in 540 participants. We observed that there is an initial decrease in ADA1 while ADA2 increased, which may suggest distinct functional roles for these proteins in the first week of life. Of note, ADA1 acts as both an ecto-enzyme and as an intracellular enzyme (10–13, 21), whereas ADA2 can bind cognate leukocyte receptors to re-shape immune polarization (14, 26, 27, 41). Intriguingly, a recent study posted to *bioRxiv* suggests that ADA2 has a role as a lysosomal DNase that degrades ligands for the cytosolic pattern recognition receptors cGAS/STING thereby limiting production of interferons (77), possibly explaining how ADA2 deficiency results in hyper-inflammation.

The plasma concentration of ADA2, and thus total ADA, transiently decreased from day of life (DOL) 0 to DOL1 likely reflecting dynamic perinatal changes. Indeed, plasma concentrations of some proteins, such as hemoglobin, are increased at delivery and decrease before reaching homeostatic levels (67–69, 78, 79). Consistent with these, when human B-cell subsets are isolated from healthy children and stimulated *ex-vivo* with oligodeoxynucleotide-2006 (ODN2006), CD40Ligand, or anti-Ig, ADA2 transcript decreased between time 0 (baseline) and Day 1, but increased again at Day 6 close to the baseline levels at time 0 (41). Interestingly, a small group of neonates in this cohort were hospitalized for a variety of suspected or confirmed diagnoses including early or late onset sepsis, pneumonia, omphalitis and impetigo (n=54); however, inclusion or exclusion of the hospitalized neonates did not change the robust and significant ontogenic patterns demonstrated during the first week of life or during the first four months of life (data not shown). In our infant cohort, after the first week of life, activity of ADA1, ADA2, and total ADA increased through the first four months of life consistent with our prior studies (3).

We examined whether a number of demographic features correlated with plasma ADA concentrations. Ethnicity can impact plasma biomarkers (80, 81) and ethnicity, language, and geography have been associated with genetic diversity in African populations (82). The concentration of ADA isoenzymes in the different ethnic sub-groups in our Gambian cohort, including Mandinka, Jola, Fula, Wolof, and Serahule were consistent throughout, suggesting conservation of the ontogeny of plasma ADA expression in these ethnic sub-groups. Given the geographic size of The Gambia and similarity in the Gambian ethnic sub-groups, we cannot draw final conclusions on the impact of ethnicity on ADA concentrations.

Sex also impacts the immune system (83) and male sex is associated with gestational complications such as failure to progress during labor (84), as well as infant susceptibility to infection and mortality (85). Furthermore, while males demonstrate increased susceptibility to a range of infections (86–89), there is an interaction between sex and age (90, 91). A small study of elderly individuals with and without stroke noted that females with a history of stroke had relatively higher plasma concentrations of ADA1 and a higher ratio of plasma ADA1/ADA2 (92). Of note, genetic polymorphisms of ADA genes associated with longevity in males but not females, though this study did not evaluate plasma ADA concentrations (93). Little is known regarding sex-based differences in ADA expression in early life. Our Gambian infant study demonstrated similar plasma concentrations of ADAs for males and females at birth with significantly elevated concentrations of ADA2 and total ADA in males by four months of life. A study of American school-aged children (mean ~8 years of age) did not reveal any significant difference in plasma ADA2 concentration based on sex (94). Further studies focusing on the phase beyond the first four months of life and before age 2 years will be important to delineate the ontogeny of sex-dependent differences in ADA2 and assess potential correlations with immunity, health, and disease given the clinical relevance of ADA2 and inflammation (32–52).

Several risk factors for infection and poor outcome including impaired neurological development have been defined for infants born between late pre-term (≥34 weeks and <37 weeks of gestation) and early term (≥37 week to <39 weeks gestation) (95, 96). Furthermore, gestational age (GA) correlates with neonatal plasma biomarkers such as hemoglobin and iron at birth (67–70). We assessed whether plasma concentrations of ADAs may correlate with GA, although by definition, we did not include any infant below 36 weeks gestation. We observed significantly elevated ADA2 and total ADA in the early term compared to full-term infants. The functional relevance of these correlations is at present unclear. Our study is limited as we did not enroll premature neonates (<36 weeks) and our patient population was not powered to study neonatal conditions (e.g. jaundice) that may impact the expression of ADAs. Future studies should characterize ADA concentrations in the premature population in relation to immunologic parameters and infection to provide further insight into the functional consequences of ADA expression in early life.

During the first two years of life, there is a gradual switch from a predominantly Th2-polarizing cytokine response toward a more balanced Th1/Th2 response (5). We previously showed that supplementing *in vitro* cultures of neonatal umbilical cord blood mononuclear cells with recombinant ADA1 enhanced TLR-mediated TNFα production (16). Moreover, addition of ADA to cultures of monocyte-derived DCs in IL-4 and GM-CSF medium enhanced DC production of TNFα, IL-6, and CXCL8 (97). To assess whether these previous *in vitro* studies may have relevance *in vivo*, we examined whether plasma concentrations of ADAs correlated with plasma cytokine or chemokine concentrations during the first week of life. Total ADA correlated with CXCL10 and TNFα but not with IFNγ or CXCL8. In assessing each of the ADAs individually, plasma ADA1 weakly correlated with plasma IL-6 and moderately with CXCL8 while ADA2 correlated positively with TNFα, IFNγ, and the IFN-inducible protein CXCL10, and negatively with IL-6 and CXCL8. Consistent with our observations, *in vitro* studies have demonstrated that stimulation of human PBMCs with IL-12, IL-18 or IFNγ, and TNFα induced expression of ADA2 (94) but not ADA1 (98). While information regarding the impact of ADA2 deficiency (DADA2) on plasma cytokines or chemokine concentrations is limited, observation of increased IL-6 plasma concentrations in a patient with ADA2-deficiency due to a missense mutation (47) is consistent with the negative correlation between ADA2 and IL-6 we observed in our infant cohort. Of note, the report on the ADA2-deficient patient after treatment with HSCT demonstrated normalization of both ADA2 and IL-6 concentrations, suggesting a close interplay between these proteins (47). Overall, there appears to be coordinated expression of ADA and cytokines, such that the cytokines induce ADAs that counter-regulate them (i.e. a potential feed-back loop).

Finally, we observed a decrease in plasma ADA2 concentrations in infants born to women ≥ 35 years of age, which significantly dropped relative to the plasma concentration in infants born to women < 35 years of age; by 6% at 1 month and by 12% at 4 months of life. This is notable as advanced maternal age is related to adverse outcomes including spontaneous abortions, preterm birth, and perinatal morbidity (71–73). Indeed, increased total ADA has been noted in maternal and umbilical cord plasma in women with pre-eclampsia (99, 100), and a genetic polymorphism of ADA1 resulting in lower ADA was associated with a maternal age-dependent lower risk for recurrent spontaneous abortions (101). While statistically significant, it is unclear if the differences observed are due to association versus a causal relationship. These observations provide a rationale for future studies to determine if the balance between ADA1 and ADA2 is relevant to maternal and perinatal health including perinatal morbidity and mortality.

Overall, our observations may have translational relevance as several of the recently described patients with ADA2 deficiency (DADA2) were diagnosed during the first two months of life after presenting with fever and/or anemia (30, 33, 39, 50). Total ADA, ADA1, and ADA2 have been explored as biomarkers (56–60, 94, 102, 103) and ADA1, as well as ADA receptor agonists/antagonists, are studied as possible vaccine adjuvants or disease-modifying drugs (20). In HIV infection, ADA1 concentration is decreased, addition of exogenous ADA1 enhances germinal center formation (102, 104), and co-immunization with HIV-1 envelope protein and plasmid-encoded ADA1 enhanced humoral immunity (105). Characterizing baseline ADA concentrations in a target population may inform translational efforts to modulate ADA-deficient states *via* administration of recombinant ADA, HSCT, or gene therapy (21, 106, 107). Moreover, baseline plasma ADA1, but not ADA2 measurements are also affected by hemolysis (98), highlighting the importance of measuring ADA1 separately from ADA2.

Our study features several strengths, including (a) a robust sample size, (b) rigorous clinical data capture, (c) a quantitative high throughput ADA assay platform that minimizes batch effects, and (d) a highly statistically significant novel observation of ontogeny-driven changes in ADA1, ADA2, and total ADA. As with all studies, our work also has some important limitations. Due to sample volume and field processing limitations, we did not measure ADA receptors, ectonucleotidases, nor the adenosine metabolite, whose half-life is fleeting. It will also be important to determine if the ADA ontogeny trajectory observed in neonates in The Gambia is generalizable to neonates in other geographic locations. Within the constraints of conducting large-scale international human neonatal studies, we described the ontogeny of ADA2 as well as total ADA and ADA1 and correlated these concentrations with those of key cytokines and chemokines. Given growing literature regarding its clinical relevance, our findings highlight the need for further study of ADA2, its mechanisms of action, genotypic variants, and associated clinical phenotypes.

In conclusion, plasma concentrations of ADAs demonstrated marked ontogenic changes across the first week and months after birth that correlated with plasma cytokine and chemokine concentrations, raising the possibility that these immunomodulatory proteins are functionally related to innate immune polarization during infancy. Given the increasing evidence of the relevance of ADA1 and ADA2 in immunity, these proteins should be further characterized as biomarkers for early life immune ontogeny as well as during responses to immune perturbation such as vaccination or infection.

## Data Availability Statement

The datasets presented in this study can be found in online repositories. The names of the repository/repositories and accession number(s) can be found below: Per NIH/NIAID guidelines, study data including plasma ADA, cytokine, and chemokine concentrations data have been publicly archived on ImmPort (https://www.immport.org/shared/home) under accession number SDY1539.

## Ethics Statement

The studies involving human participants were reviewed and approved by The Gambia Government/Medical Research Council Unit The Gambia Joint Ethics Committee and The Boston Children’s Hospital Institutional Review Board. The patients/participants provided their written informed consent to participate in this study.

## Author Contributions

Manuscript was drafted by OO, KS, and AP. OL, BK, and TK designed the EPIC study. AO provided expert input on study design and statistical analyses; the members of EPIC listed below assisted with sample processing, clinical data capture, data quality control, and database management. MP developed and validated the ADA assay used and helped edit the manuscript. OI and JP provided support in conducting the ADA assays. Ol provided support to the *Precision Vaccines Program* Data Management Core, led by AO, for quality assurance of the clinical and biomarker data. All authors contributed to the article and approved the submitted version.

The Expanded Program on Immunization (EPIC) Consortium consists of the following member (in addition to the main authors) listed alphabetically: Nelly Amenyogbe, Asimenia Angelidou, Winnie Bao, Rym Ben-Othman Tue B. Bennike, Morten Bjerregaard-Andersen, Ryan R. Brinkman, Byron Brook, Kendyll Burnell, Bing Cai, Abhinav Checkervarty, Virginia Chen, Mitchell Cooney, Momoudou Cox, Alansana Darboe, Bhavjinder K. Dhillon, Tida Dibassey, Joann Diray-Arce, Reza Falsafi, Benoit Fatou, Rebecca Ford, Freddy Francis, Christian N Golding, Robert E.W. Hancock, Danny J Harbeson, Daniel He, Samuel H. Hinshaw, Joe Huang, Abdulazeez Imam, Wendy Kirarock, Ken Kraft, Kristina Lindberg Larsen, Amy H. Lee, Aaron Liu, Mark Liu, Mehrnoush Malek, Arnaud Marchant, Geraldine Masiria, John Paul Matlam, Kerry McEnaney, Sebastiano Montante, Elena Morrocchi, Jorjoh Ndure, Jainaba Njie-Jobe, Edward Parker, William S. Pomat, Shun Rao, Peter C. Richmond, Elishia Roberts, Gerard Saleu, Lilica Sanca, Guzman Sanchez-Schmitz, Frederik Schaltz-Buchholzer, Casey P Shannon, Amrit Singh, Maren Smith, Hanno Steen, Julia Strandmark, Scott J Tebbutt, Anita H.J. van den Biggelaar, Simon van Haren, Natallia Varankovich, Sofia Vignolo, Diana Vo, Oghenebrume Wariri.

## Funding

This work was supported by funds from the National Institute of Health (NIH), National Institute of Allergy and Infectious Disease (NIAID) division as part of the Human Immunology Project Consortium (U19AI118608) to OL, and funds from the Boston Children’s Global Health Program and an NIH Loan Repayment Program award, the National Institute on Minority Health and Health Disparities (NIMHD) to OO. The *Precision Vaccines Program* is supported in part by the Department of Pediatrics and Chief Scientific Officer of Boston Children’s Hospital. The content is solely the responsibility of the authors and does not necessarily represent the official views of the National Institutes of Health or Boston Children’s Hospital.

## Conflict of Interest

OL is a named inventor on several patents related to microphysiologic platforms that model human immunity *in vitro*, anti-infective proteins, and vaccine adjuvants.

The remaining authors declare that the research was conducted in the absence of any commercial or financial relationships that could be construed as a potential conflict of interest.

## Footnotes

1 In EPIC002, all maternal participants are age 18 and above.

## Abbreviations

ADA, adenosine deaminase; cGAS, cyclic guanosine monophosphate–adenosine monophosphate; CXCL, chemokine (C-X-C motif) ligand; DOL, Day of Life; EPIC, Expanded Program on Immunization Consortium; EHNA, Erythro-9-(2-hydroxy-3-nonyl) adenine; GT, Gene therapy; HIPC, Human Immune Project Consortium; HSCT, Hematopoietic stem cell transplantation; Ig, immunoglobulin; IL, Interleukin; IP, IFNγ-Inducible Protein; kDa, Kilo Daltons; NK, Natural Killer; rADA, recombinant ADA; STING, stimulator of interferon genes; and Th, T-helper.
