# Supplementary material for: Plasma Adenosine Deaminase (ADA)-1 and -2 Demonstrate Robust Ontogeny Across the First Four Months of Human Life
Source: Front Immunol. 2021 May 27;12:578700. doi: 10.3389/fimmu.2021.578700 (PMC8190399; doi:10.3389/fimmu.2021.578700)
Supplement: Supplementary file 1 [file DataSheet_1.docx]

Supplementary Material

# Supplementary Data

All supplementary data will be uploaded along with data from the primary manuscript to ImmPort (https://www.immport.org/shared/home) per NIAID data sharing policy for the Human Immunology Project Consortium study under accession number SDY1539.

# Supplementary Figures and Tables

## Supplementary Figures


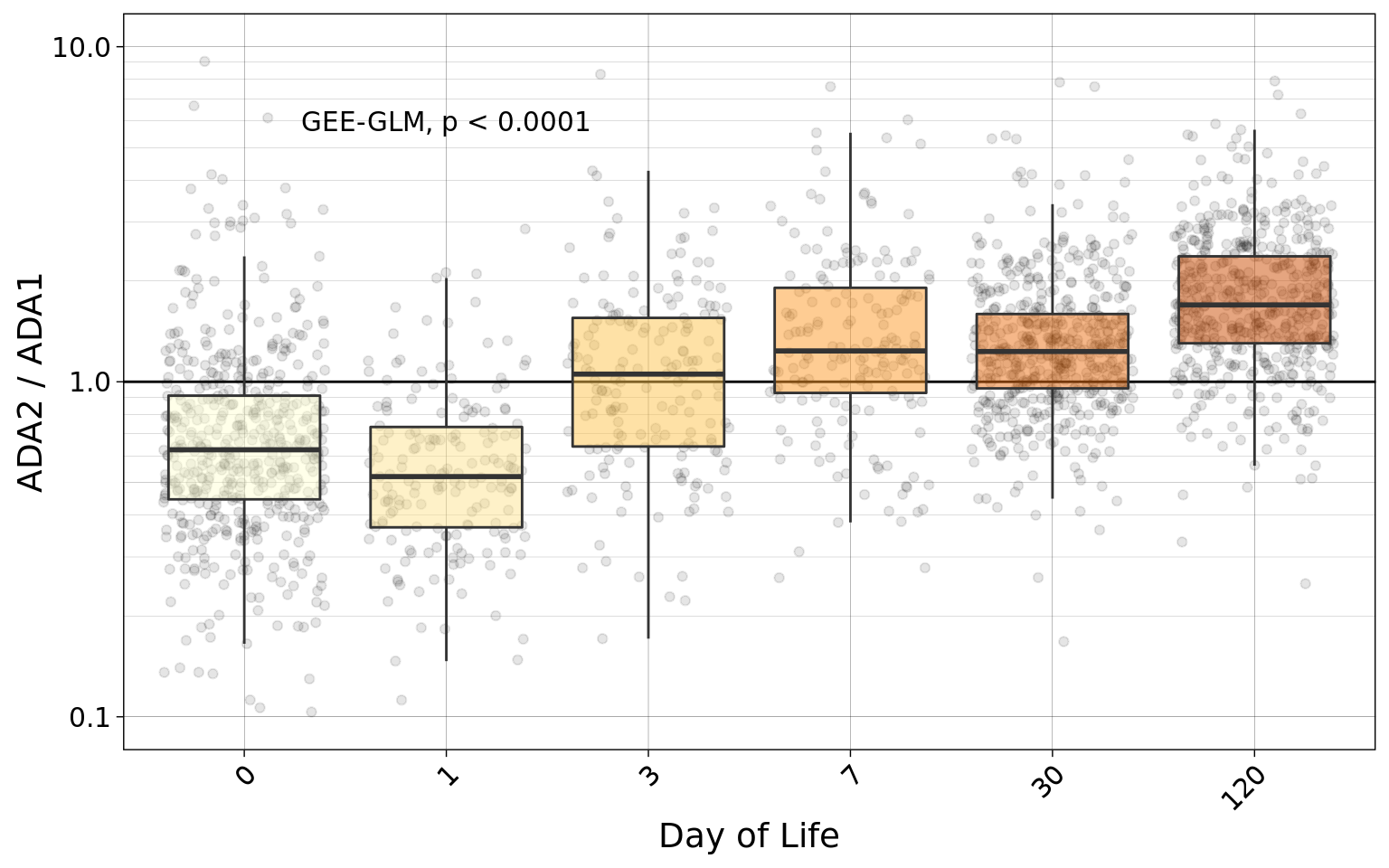


**Supplementary Figure 1.** **Relative ratio of plasma ADA1 to ADA2 concentrations increases during the first four months of life.** Ratio of ADA1 and ADA2 in the Gambian cohort demonstrated an increase of plasma ADA2 concentration relative to plasma ADA1 during the first four months of life (n = 514-525 for DOL 0, 30, and 128; n = 173-176 for DOL 1, 3, 7).  Statistics fit a GEE-GLM to log_10_ (isoform ratio) with Visit and Day of Life (DOL 1,3 or 7) as predictors, using Gaussian distribution, identity link function, and exchangeable covariance structure. Deviance analysis was conducted by comparing GEE to a null model, and p-values were found using the Wald statistic.

(A)


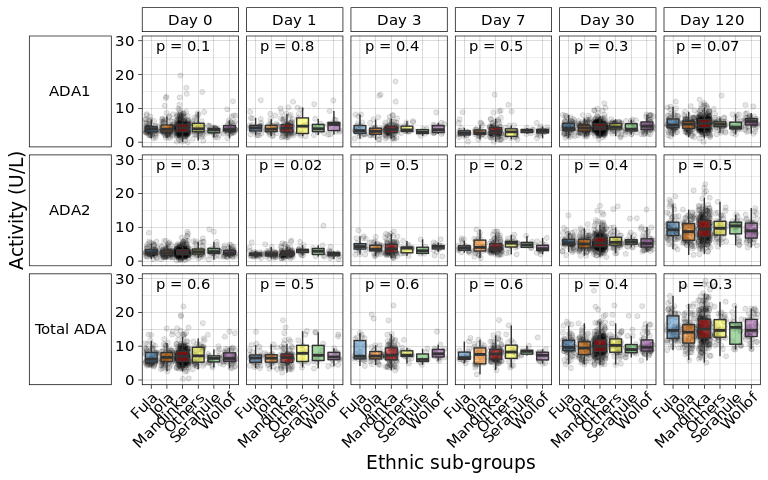


(B)


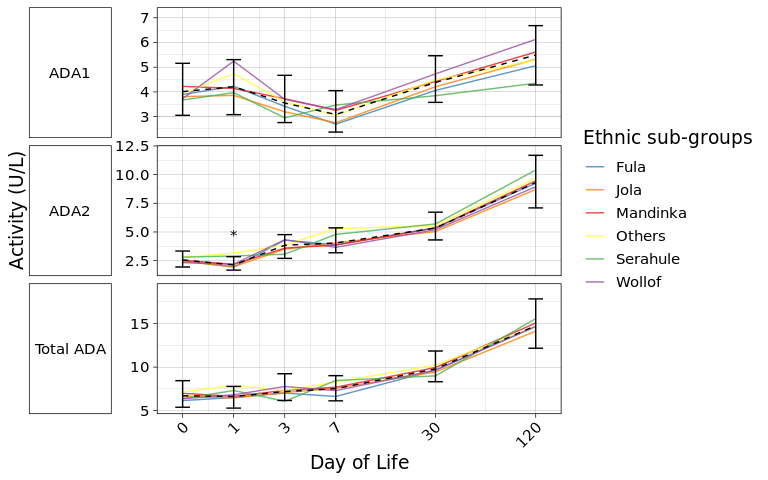


**Supplementary Figure 2.** **Gambian ethnic sub-groups share a similar course of ADA ontogeny.** Measurement of plasma ADA1, ADA2 and Total ADA in the Gambian cohort showed that there was no difference in ADA1, ADA2, and total ADA concentrations between different Ethnic sub-groups (a) and all sub-groups follow a similar pattern (b) (n = 22-269 per group) except Day of Life 1 where the Others group may have slightly higher ADA2 levels (p=0.02). Dotted line and error bars represent median and IQR for all participants, respectively. Statistics used Kruskal-Wallis.

(A)


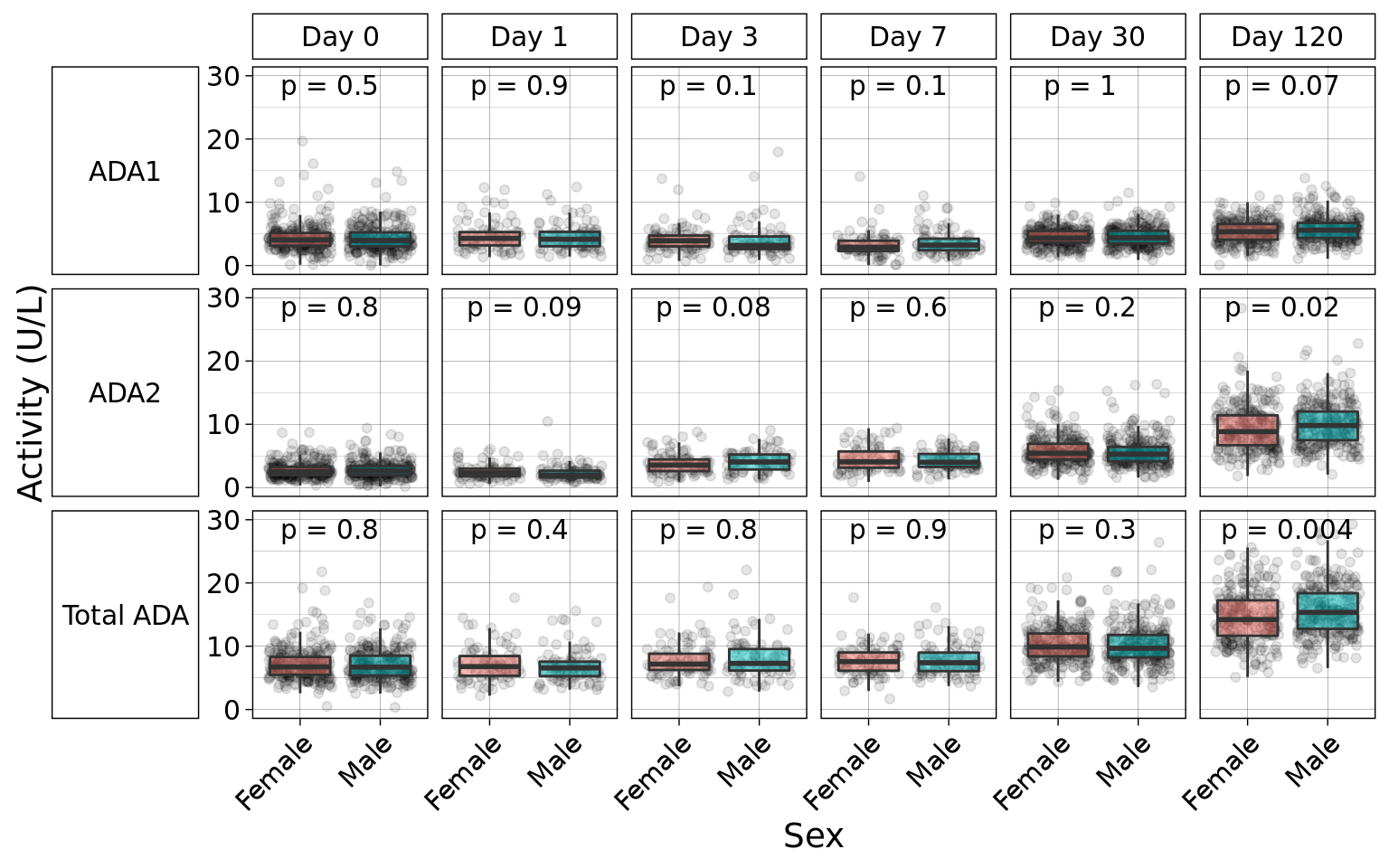


(B)


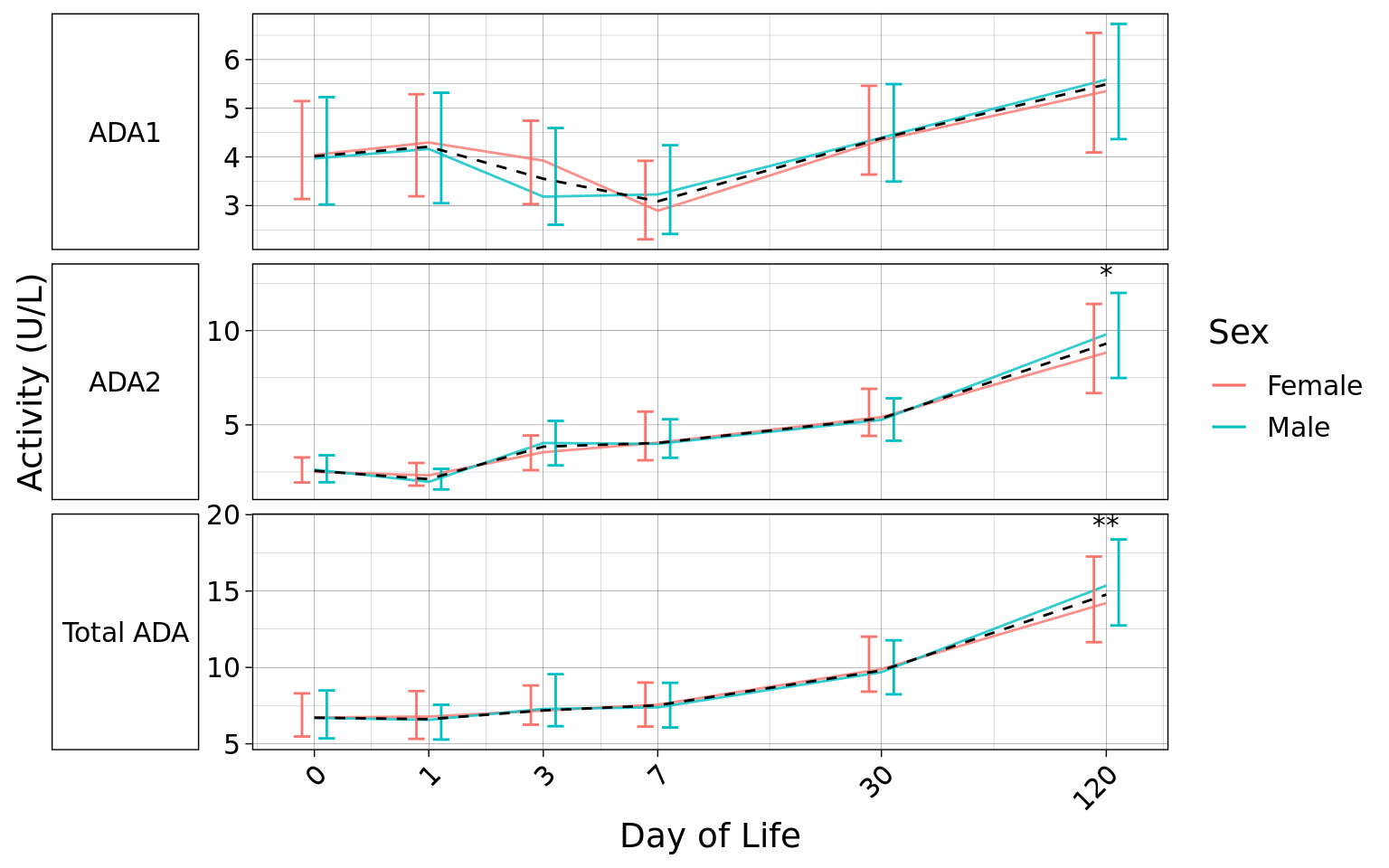


**Supplementary Figure 3.** **Males exhibit higher plasma concentrations of ADA2 and Total ADA activity measured *ex vivo* at the fourth month of life.** Measurement of plasma ADA1, ADA2, and Total ADA in the Gambian cohort showed elevated concentrations in ADA2 (p=0.02), and Total ADA (p=0.004) but not ADA1 (p = 0.07) in males compared to females seen at four months of life but not at earlier timepoints (n = 81-264 per group). Dotted line and error bars represent median and IQR for all participants, respectively. Statistics used Wilcoxon.

(A)


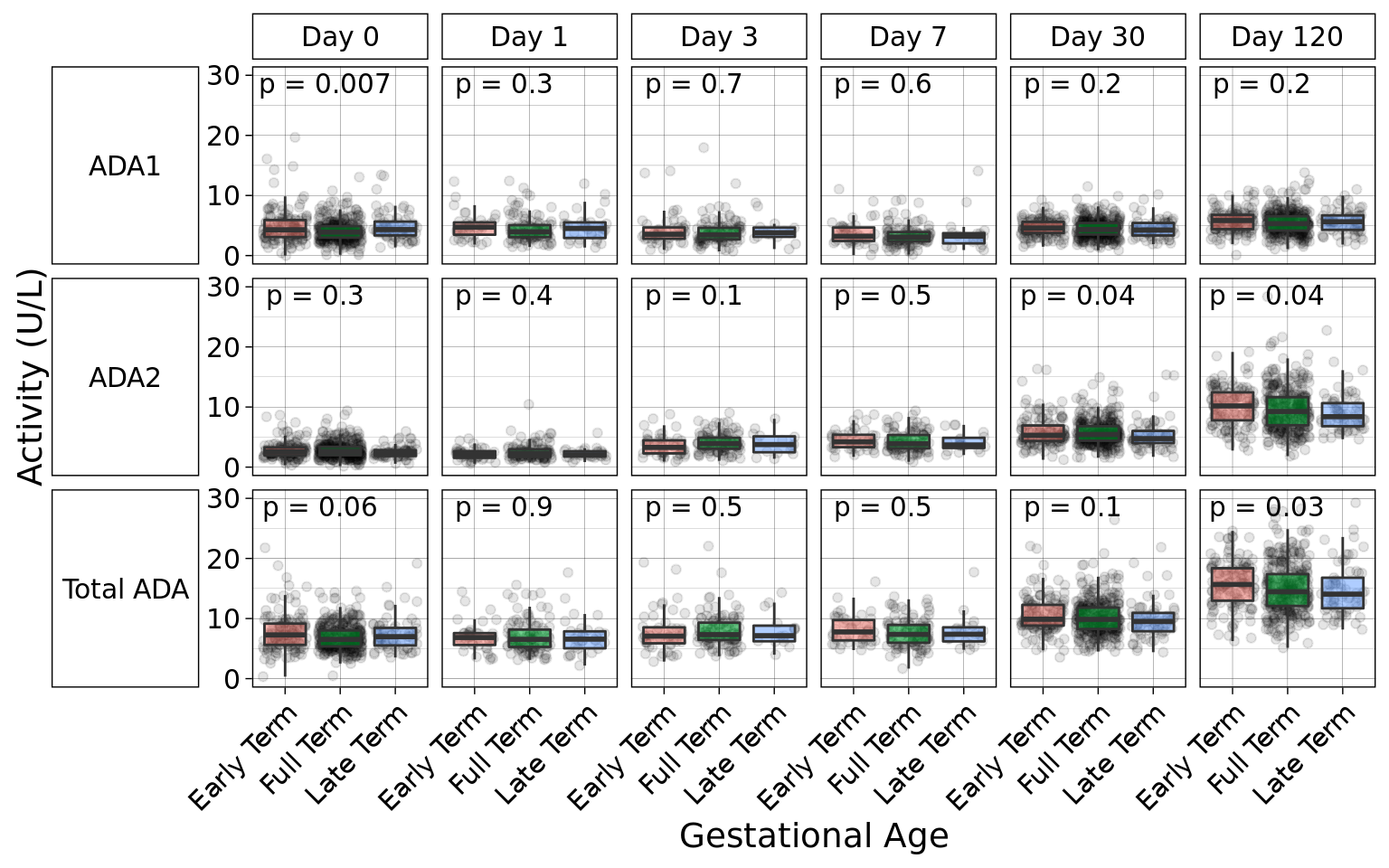


(B)


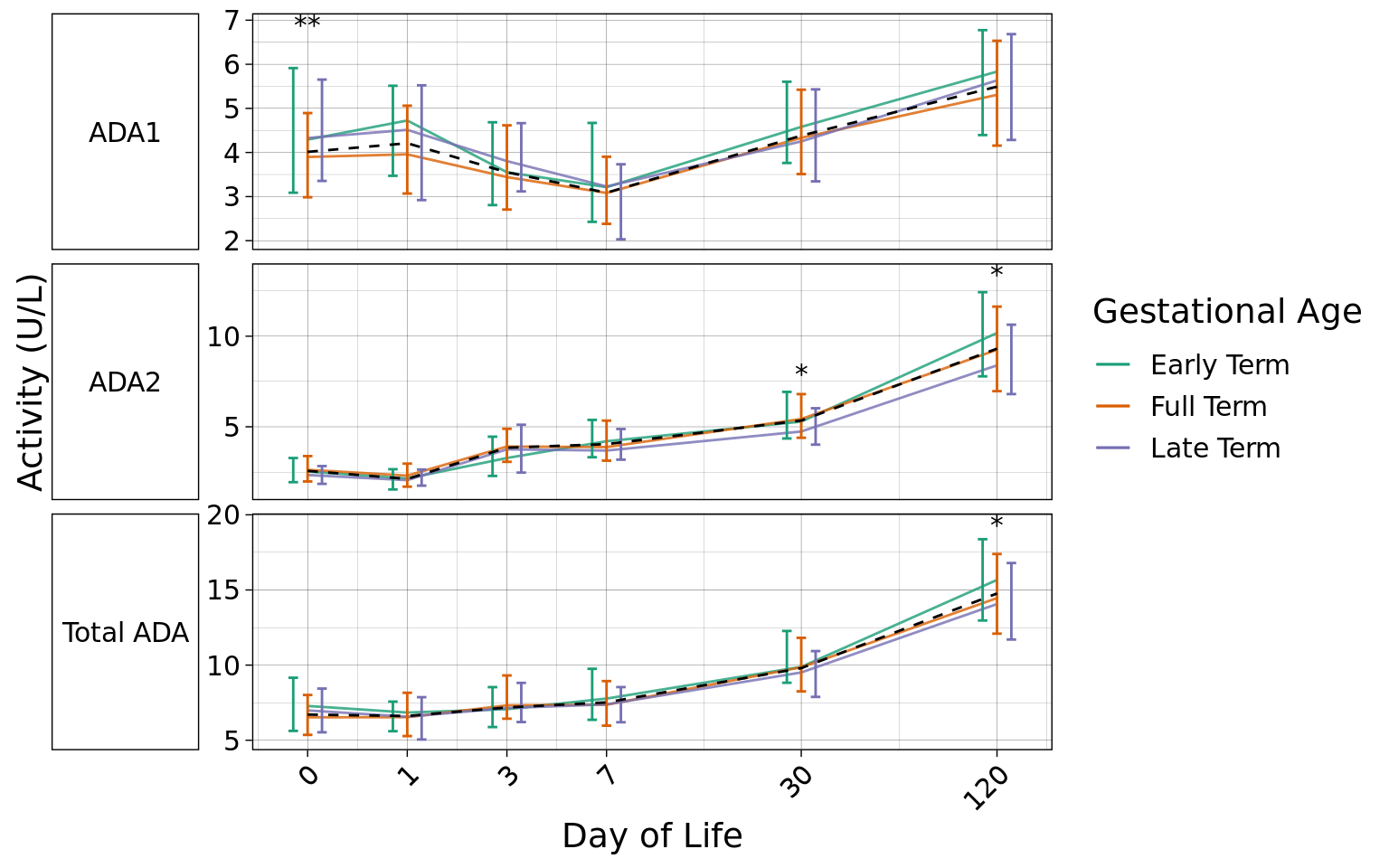


**Supplementary Figure 4.** **Gestational age and ADA isotype ontogeny.** Measurement of plasma ADA1, ADA2, and Total ADA concentrations over time in the Gambian cohort showed significantly elevated concentration of ADA2 and total ADA in the early term compared to full-term infants at later time points (n = 39-133 early term infants per time point, n = 107-330 full term infants per time point, n = 15-65 late term infants per time point). Dotted line and error bars represent median and IQR for all participants, respectively. Statistics used Kruskal-Wallis

(A)


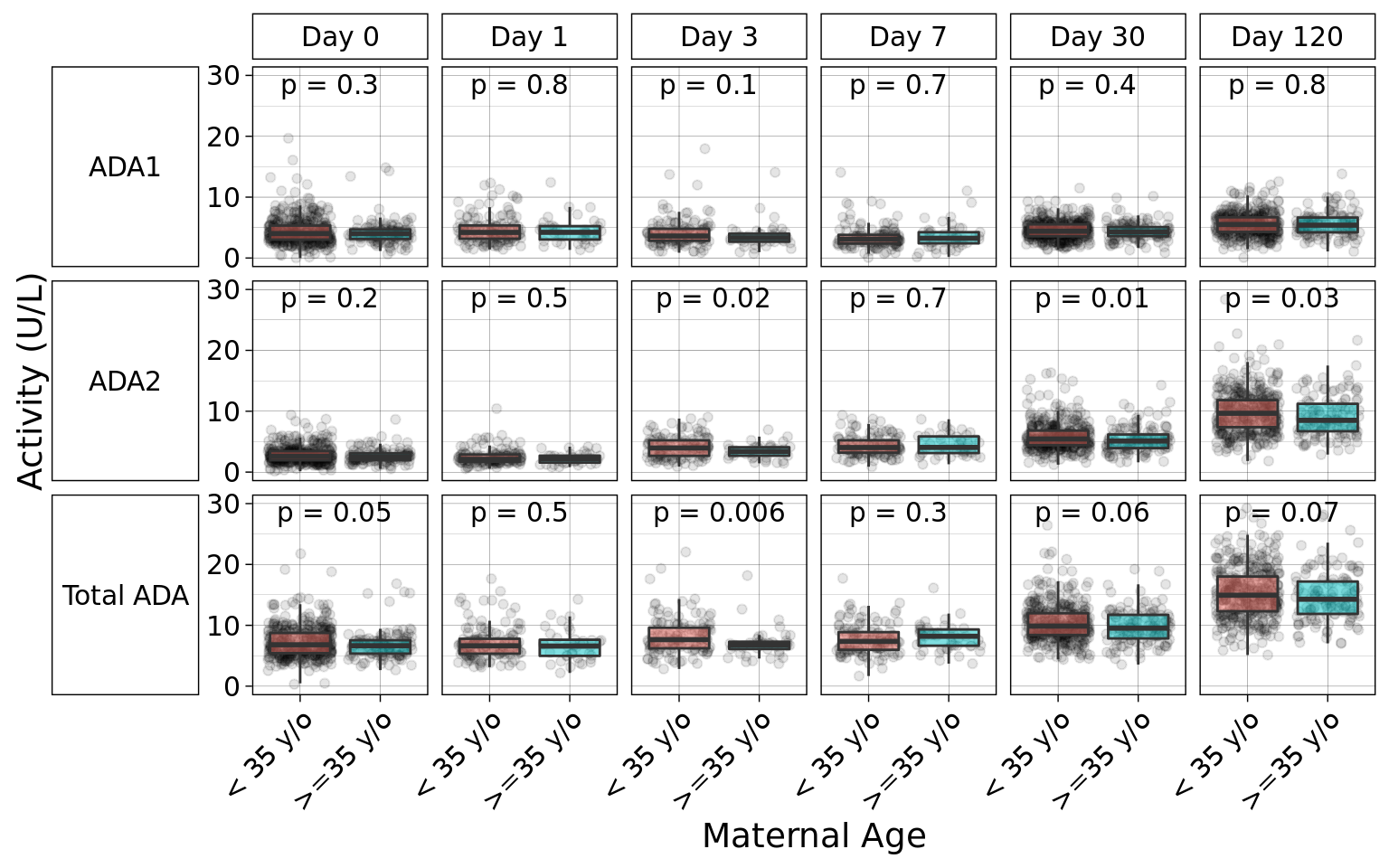


(B)


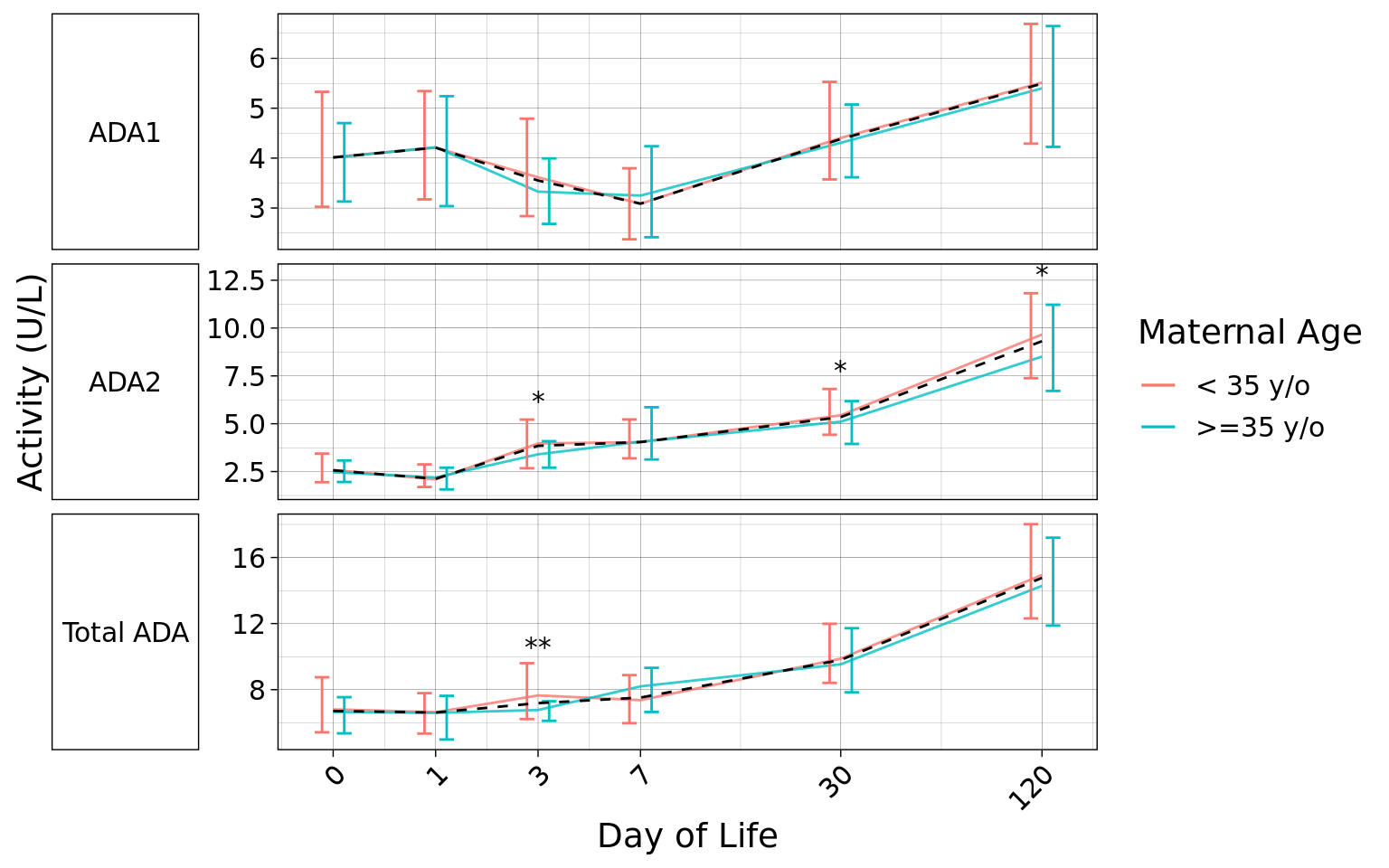


**Supplementary Figure 5.** **Greater maternal age is associated with lower ADA2 plasma concentrations during the first four months of life.** Measurement of plasma ADA1, ADA2, and Total ADA at during month 1 and month 4 in the Gambian cohort showed significantly elevated Total ADA during Day of Life (DOL) 3, and ADA2 during DOL 3, 30, and 120 (n = 131- 400 infants of mothers <35 years of age, n = 38-126 infants of mothers >35 years of age). Dotted line and error bars represent median and IQR for all participants, respectively. Statistics used Wilcoxon.
